# Organ-Preserving Gastric Wedge Resection for a Giant Posterior Gastric Gastrointestinal Stromal Tumor: A Case Report

**DOI:** 10.7759/cureus.111233

**Published:** 2026-06-21

**Authors:** Osmar Kengi Yagi, Luis Francisco Llerena Freire, Marcus Fernando Kodama Pertille Ramos, Ulysses Ribeiro Junior

**Affiliations:** 1 Digestive Surgery, Hospital das Clínicas da Faculdade de Medicina da Universidade de São Paulo, Sao Paulo, BRA; 2 Digestive Surgery, Instituto do Câncer do Estado de São Paulo, Sao Paulo , BRA

**Keywords:** gastric neoplasms, gastrointestinal stromal tumor, gist, organ-preserving surgery, wedge resection

## Abstract

Gastrointestinal stromal tumors (GISTs) are the most common mesenchymal neoplasms of the gastrointestinal tract, with the stomach representing the predominant site of origin. Although complete surgical resection remains the standard treatment for localized disease, the management of giant gastric GISTs can be challenging because of their size, anatomical relationships, and the risk of tumor rupture during surgery. We report the successful organ-preserving resection of a giant posterior gastric GIST using a wedge resection technique.

A 49-year-old woman presented with a two-year history of abdominal pain, postprandial discomfort, abdominal distension, nausea, and vomiting. Contrast-enhanced computed tomography revealed a large lobulated mass measuring 14.0 × 12.0 × 11.0 cm, closely associated with the stomach. Endoscopic ultrasound demonstrated a heterogeneous hypoechoic lesion originating from the muscularis propria, and fine-needle aspiration findings were consistent with GIST. Exploratory laparotomy through a midline incision identified a giant exophytic tumor arising from the posterior gastric wall without evidence of metastatic disease or adjacent organ invasion. Complete resection was achieved by gastric wedge resection using a 75-mm linear stapler, avoiding formal gastrectomy. The operative time was 120 minutes, estimated blood loss was 100 mL, and no intraoperative complications occurred. The patient was discharged on postoperative day 3. Histopathological examination demonstrated an 11.1 × 10.8 × 8.5 cm spindle-cell GIST positive for CD117 and DOG1, with low mitotic activity, negative surgical margins, and pathological stage pT4. According to the modified NIH (Joensuu) risk stratification criteria, the tumor was classified as having a moderate risk of recurrence.

Giant posterior gastric GISTs can be safely managed with organ-preserving wedge resection when complete oncologic excision is feasible. Careful surgical planning and meticulous handling to avoid tumor rupture may allow excellent short-term outcomes while preserving gastric anatomy and function.

## Introduction

Gastrointestinal stromal tumors (GISTs) are the most common mesenchymal neoplasms of the gastrointestinal tract, although they account for less than 1% of all gastrointestinal malignancies. They are believed to originate from the interstitial cells of Cajal or their precursors and are characterized by activating mutations in the KIT or platelet-derived growth factor receptor alpha (PDGFRA) genes, resulting in constitutive tyrosine kinase signaling and uncontrolled cellular proliferation [1,2].

The stomach is the most frequent site of origin, accounting for approximately 60% of all GISTs, followed by the small intestine, colon, rectum, and esophagus [3]. Clinical presentation is highly variable and depends on tumor size, location, and growth pattern. Small lesions are often asymptomatic and detected incidentally, whereas larger tumors may present with abdominal pain, gastrointestinal bleeding, early satiety, a palpable abdominal mass, or nonspecific gastrointestinal symptoms [4].

Cross-sectional imaging and endoscopic ultrasound (EUS) play a central role in the diagnostic evaluation of suspected GISTs. Computed tomography (CT) is considered the imaging modality of choice for staging and surgical planning, while EUS-guided fine-needle aspiration (FNA) enables preoperative tissue diagnosis and immunohistochemical characterization [5]. The diagnosis is typically confirmed by positive immunohistochemical staining for KIT (CD117) and DOG1, which are expressed in the majority of cases [6].

Complete surgical resection with negative microscopic margins (R0 resection) remains the cornerstone of treatment for localized, resectable GISTs. Unlike gastric adenocarcinoma, lymphatic spread is uncommon, and routine lymphadenectomy is generally not required. Consequently, organ-preserving resections are preferred whenever technically feasible [7]. Current surgical principles emphasize complete tumor removal while avoiding intraoperative tumor rupture, which is recognized as one of the strongest predictors of recurrence and adverse oncologic outcomes [8].

Although tumors larger than 10 cm are commonly classified as giant GISTs, they remain relatively uncommon in contemporary practice due to the widespread use of abdominal imaging and earlier diagnosis. Giant gastric GISTs may pose significant technical challenges because of their size, distortion of surrounding anatomy, and concerns regarding involvement of adjacent organs. Nevertheless, in selected cases, limited gastric resections can achieve adequate oncologic clearance while preserving gastric function and avoiding unnecessary gastrectomy [9].

We report the case of a giant posterior gastric GIST measuring 14 cm on preoperative imaging that was successfully managed through exploratory laparotomy and organ-preserving gastric wedge resection using a linear stapler, achieving complete oncologic resection without tumor rupture.

Posterior gastric wall GISTs present unique surgical challenges because of their limited accessibility, close relationship with adjacent structures such as the pancreas and splenic vessels, and the increased risk of tumor rupture during mobilization. Historically, giant gastric GISTs were frequently treated with formal gastric resections to ensure complete tumor removal. However, growing evidence has demonstrated that when negative margins can be achieved without tumor rupture, organ-preserving procedures such as wedge resection can provide adequate oncologic control while preserving gastric anatomy and function. This evolution toward function-preserving surgery has become increasingly important in the management of selected patients with large localized gastric GISTs.

## Case presentation

A 49-year-old woman presented with a two-year history of progressive abdominal pain and abdominal distension associated with postprandial worsening. The symptoms were accompanied by nausea and vomiting. One month before admission, she reported the onset of daily diarrhea with three to four episodes per day.

Contrast-enhanced CT of the abdomen and pelvis demonstrated a large lobulated solid mass measuring 14.0 × 12.0 × 11.0 cm located in the epigastrium and left upper abdomen. The lesion was closely related to the lesser curvature of the stomach, omentum, and jejunal loops, causing displacement of the descending colon. No distant metastases, enlarged lymph nodes, or evidence of peritoneal disease were identified (Figure 1).

**Figure 1:**
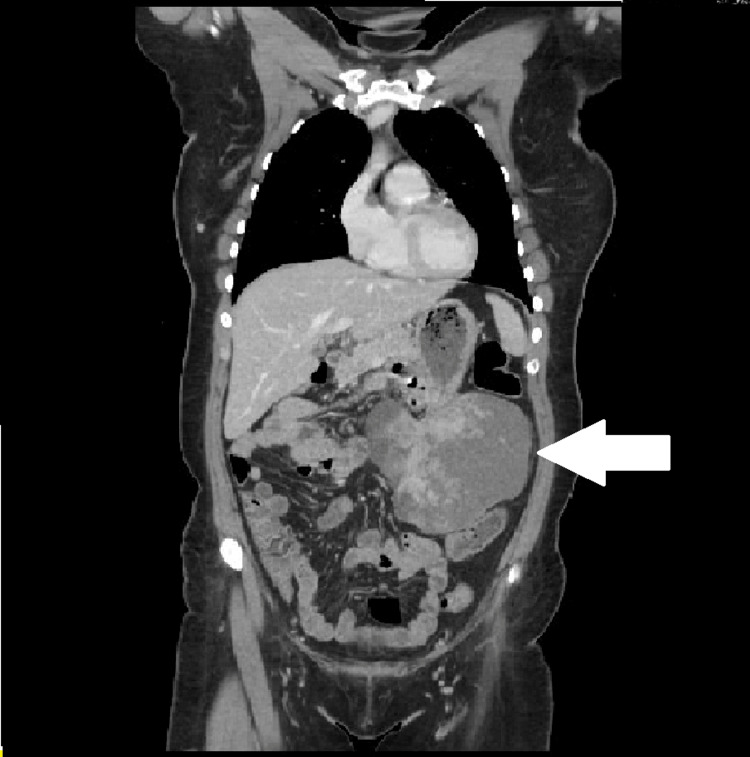
Coronal contrast-enhanced CT images showing a large gastrointestinal stromal tumor (GIST) arising from the stomach (white arrow).

EUS revealed a heterogeneous hypoechoic lesion measuring approximately 13 × 8 cm, with irregular borders, intralesional vascularity, and focal necrotic areas. The lesion appeared to arise from the muscularis propria layer of the gastric wall. FNA demonstrated findings compatible with a GIST (Figure 2).

**Figure 2:**
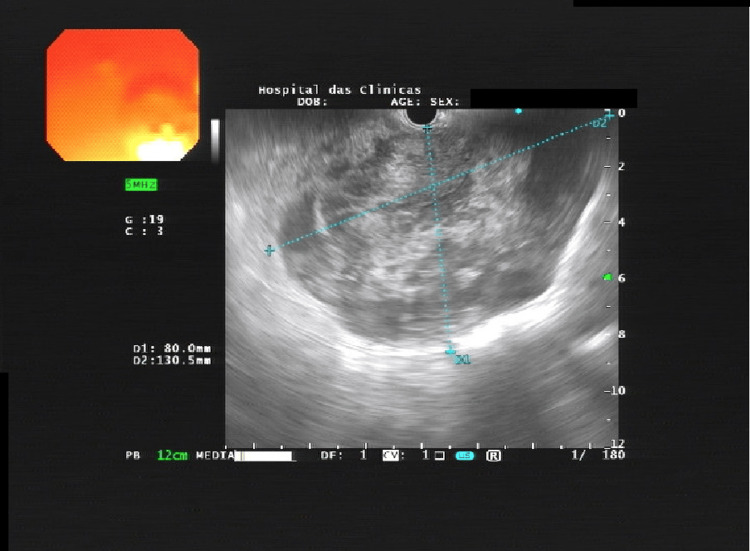
Endoscopic ultrasound demonstrating a heterogeneous lesion arising from the muscularis propria.

Following multidisciplinary discussion, surgical resection was indicated. On April 15, 2026, the patient underwent exploratory laparotomy through a midline supra- and infraumbilical incision. Intraoperatively, a giant exophytic gastric tumor measuring approximately 13 cm was identified arising from the posterior wall of the gastric body. The lesion was well circumscribed, without evidence of local invasion of adjacent organs, peritoneal dissemination, or liver metastases. Particular care was taken during mobilization to avoid tumor rupture, which did not occur (Figure 3).

**Figure 3:**
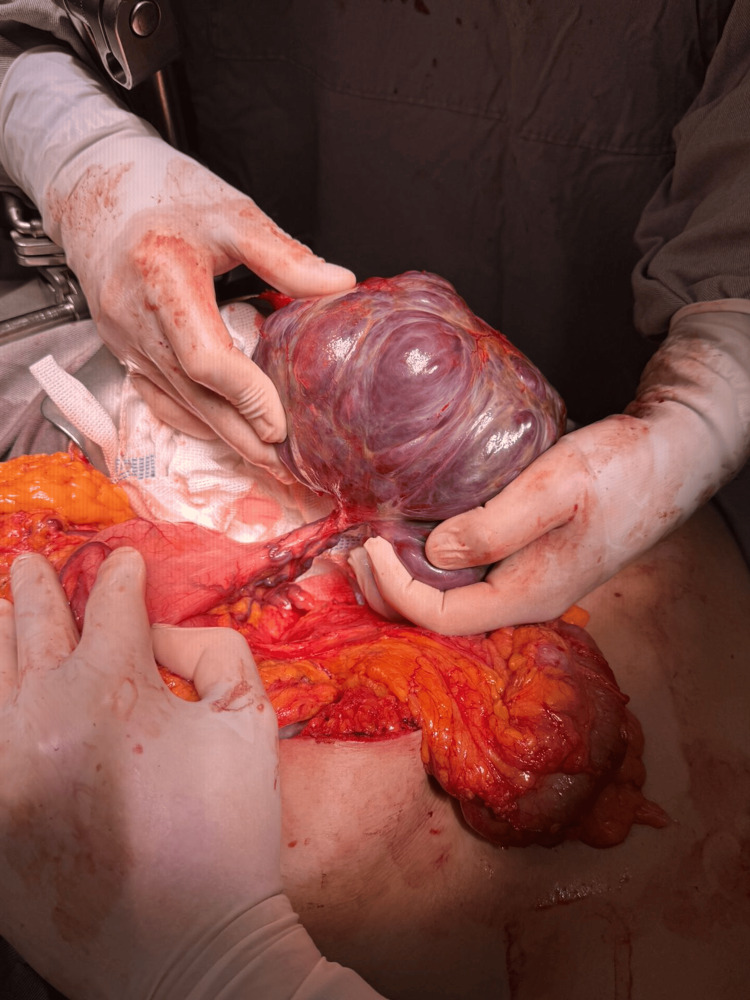
Intraoperative view of the giant exophytic posterior gastric gastrointestinal stromal tumor.

Complete resection was achieved by wedge gastric resection using a 75-mm linear stapler. Grossly negative margins were obtained, and gastric preservation was possible without the need for partial or total gastrectomy (Figure 4).

**Figure 4:**
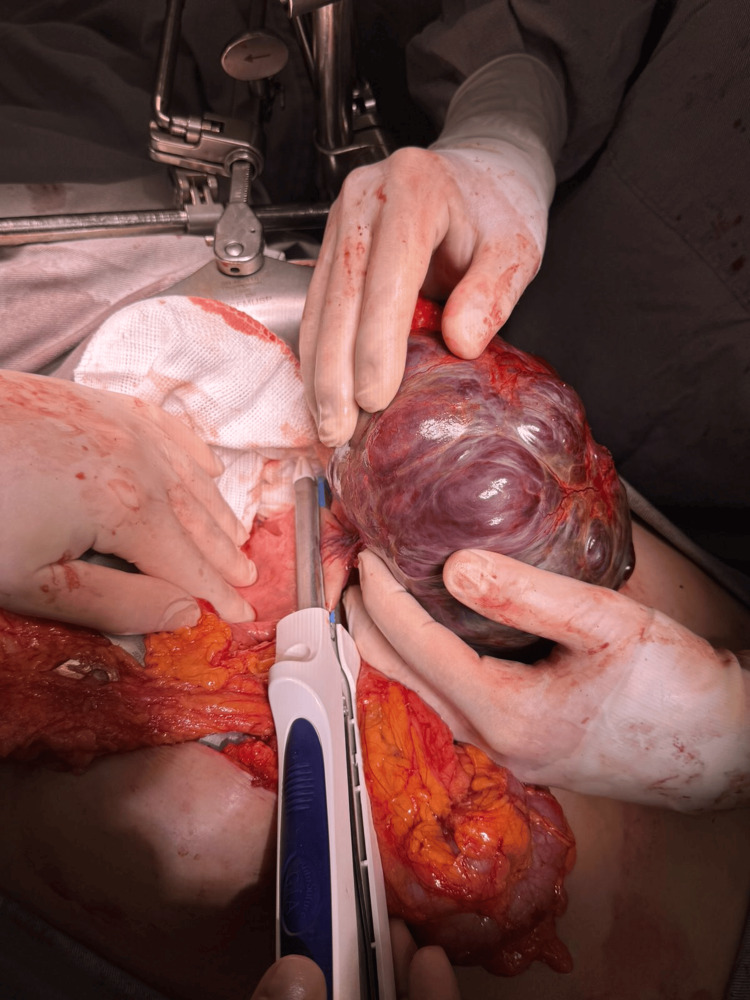
Stapled wedge gastric resection using a 75-mm linear stapler.

Operative time was 120 minutes, and estimated blood loss was approximately 100 mL. No intraoperative complications occurred. The postoperative course was uneventful. Oral intake was progressively resumed, pain was adequately controlled, and the patient was discharged home on postoperative day three in good clinical condition. Pathological examination demonstrated a lobulated tumor weighing 808 g and measuring 11.1 × 10.8 × 8.5 cm (Figure 5).

**Figure 5:**
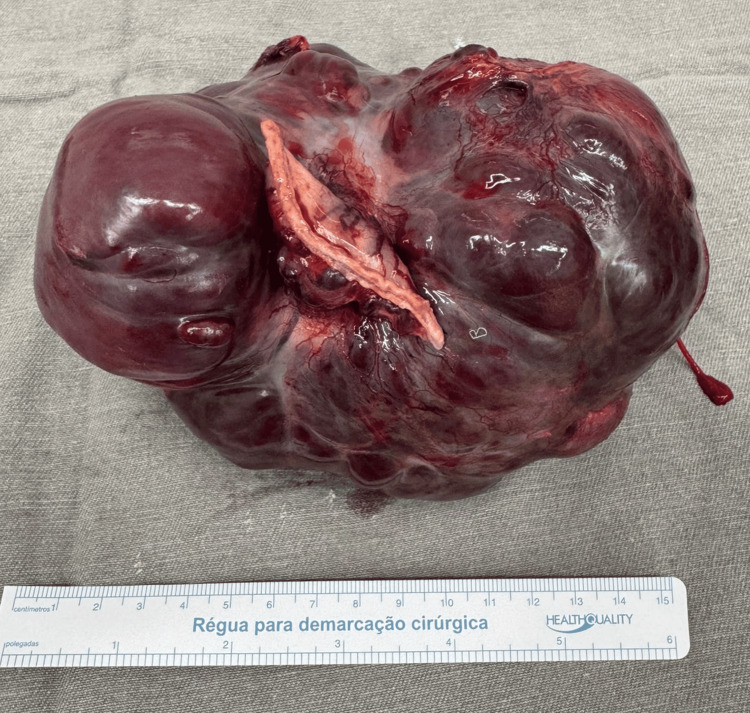
Gross specimen showing the resected giant gastric GIST measuring 11.1 cm and weighing 808 g. GIST: gastrointestinal stromal tumor.

Histologically, the lesion was composed of spindle cells and was centered in the muscularis propria. Immunohistochemical analysis revealed positivity for CD117 and DOG1 were positive, confirming the diagnosis of GIST. Desmin, CD34, and S100 were negative, helping exclude alternative mesenchymal neoplasms such as leiomyoma, leiomyosarcoma, and schwannoma. Ki-67 proliferation index was approximately 8%.

Mitotic activity was low, with up to one mitosis per 5 mm². No tumor necrosis was identified. Surgical margins were free of neoplasia (R0 resection). According to the American Joint Committee on Cancer (AJCC) 8th edition staging system, the tumor was classified as pT4 and low grade (G1). According to the modified NIH (Joensuu) risk stratification system, it was classified as having a moderate risk of recurrence.

At the latest follow-up, six months after surgery, the patient remains under regular surveillance, which consisted of periodic clinical evaluation and abdominal imaging. The patient tolerated a regular diet, maintained stable body weight, and reported no symptoms of gastric dysfunction during follow-up.

## Discussion

GISTs are the most common mesenchymal neoplasms of the gastrointestinal tract, although they remain relatively rare compared with epithelial gastrointestinal malignancies. The stomach is the primary site of origin in approximately 60% of cases and generally exhibits a more favorable biological behavior than small intestinal GISTs of similar size and mitotic activity [1,3].

The present case illustrates several clinically relevant aspects of contemporary GIST management. First, despite its considerable size, the tumor remained localized without evidence of nodal involvement, distant metastasis, or invasion of adjacent organs. This observation is consistent with the biological behavior of many gastric GISTs, which often demonstrate an expansive rather than infiltrative growth pattern and may reach substantial dimensions before diagnosis [4,9].

Tumor size is one of the most important prognostic factors in GIST and is incorporated into the most widely used risk stratification systems. Lesions larger than 10 cm are generally classified as giant GISTs and are associated with an increased risk of recurrence. However, prognosis depends not only on tumor size but also on mitotic activity, anatomical location, and the occurrence of tumor rupture [10]. In the present case, despite the large tumor size, the mitotic index was low (≤1 mitosis/5 mm²), no tumor necrosis was identified, and tumor rupture was avoided, factors that likely contributed to the moderate-risk classification observed on final pathological evaluation.

Cross-sectional imaging and EUS played a fundamental role in the preoperative assessment. Computed tomography remains the imaging modality of choice for diagnosis, staging, and surgical planning because it allows accurate evaluation of tumor size, anatomical relationships, and metastatic disease [5]. In our patient, CT demonstrated a large mass closely associated with the stomach but without evidence of distant spread, supporting a curative surgical approach. Furthermore, EUS-guided FNA provided a preoperative diagnosis consistent with GIST, facilitating multidisciplinary planning and excluding alternative mesenchymal or retroperitoneal neoplasms [5,6].

Current international guidelines recommend complete surgical resection with microscopically negative margins (R0 resection) as the standard treatment for localized, resectable GISTs [7,8]. Unlike gastric adenocarcinoma, routine lymphadenectomy is not recommended because lymph node metastases are exceedingly rare in adult patients with GIST. Therefore, the primary surgical objective is complete tumor removal while preserving organ function whenever feasible [7].

One of the most important oncologic principles in GIST surgery is the avoidance of tumor rupture. Several studies have demonstrated that intraoperative rupture is associated with a markedly increased risk of peritoneal dissemination and recurrence, to the extent that some authors consider rupture equivalent to metastatic disease in terms of recurrence risk [11]. For this reason, meticulous dissection and gentle tumor handling were emphasized throughout the procedure. The intact pseudocapsule observed intraoperatively and the absence of tumor rupture represented important technical achievements that likely contributed to the favorable postoperative prognosis.

The optimal extent of gastric resection for large GISTs remains a matter of debate. Historically, giant tumors often prompted formal gastrectomies because of concerns regarding margin status and local extension. However, several contemporary studies have demonstrated that wide margins are not required, provided that complete microscopic excision can be achieved [12]. Because GISTs rarely exhibit infiltrative growth patterns, limited gastric resections may provide oncologic outcomes comparable to those of more extensive procedures while preserving gastric anatomy and function [13].

In the present case, the tumor originated from the posterior wall of the gastric body and demonstrated a predominantly exophytic growth pattern with a relatively limited gastric attachment. These characteristics allowed gastric wedge resection using a 75-mm linear stapler without the need for subtotal or total gastrectomy. Organ-preserving surgery offers several potential advantages, including shorter operative times, lower postoperative morbidity, faster recovery, preservation of gastric reservoir function, and improved postoperative quality of life [12,13]. The patient’s uneventful recovery and discharge on postoperative day three further support the feasibility of this approach in appropriately selected patients.

The choice between open and laparoscopic resection for gastric GISTs remains dependent on tumor size, location, and surgeon experience. In the present case, an open approach was preferred because of the tumor’s large size, posterior gastric wall origin, and the need to minimize the risk of intraoperative rupture. Open surgery provided optimal exposure and facilitated safe manipulation and complete oncologic resection [12]. 

Another noteworthy aspect of this case is the discrepancy between tumor size and biological aggressiveness. Although the lesion measured 14 cm radiologically and 11.1 cm on pathological examination, the mitotic index remained low and the Ki-67 proliferation index was approximately 8%. Previous studies have demonstrated that tumor size alone should not be used as a surrogate marker of aggressive behavior, as some large gastric GISTs may follow a relatively indolent course when mitotic activity is limited [14]. This reinforces the importance of integrating pathological and molecular features into risk assessment rather than relying solely on tumor dimensions. The discrepancy between radiologic and pathologic measurements may be explained by differences in measurement techniques, specimen handling after resection, tumor collapse following removal, and formalin fixation, all of which are known to reduce final specimen dimensions. Similar differences between imaging-based and pathological measurements have been reported in other large soft tissue tumors and GIST [13].

Although the tumor exceeded 10 cm in size, the low mitotic index, absence of tumor rupture, and moderate-risk classification influenced postoperative management. Adjuvant imatinib is generally recommended for high-risk GISTs, whereas its role in moderate-risk tumors remains less clearly defined. In this case, surveillance following complete R0 resection was considered the most appropriate management strategy [15].

Finally, this case highlights the importance of individualized surgical decision-making. Current guidelines emphasize tailoring the surgical strategy according to tumor location, growth pattern, and resectability rather than adopting a uniform surgical approach [7,8]. Although neoadjuvant imatinib may be considered in selected patients with marginally resectable tumors or when organ preservation would otherwise be impossible, upfront surgery remains appropriate for localized lesions amenable to complete resection [15]. In our patient, complete resection was technically feasible at presentation, making primary surgical treatment the most reasonable management strategy. Molecular testing for KIT and PDGFRA mutations was not available in this case and, therefore, could not be incorporated into the risk assessment or therapeutic decision-making process. This represents a limitation of the present report. 

This report is limited by its single-case design and relatively short follow-up period. Therefore, the findings should be interpreted with caution and may not be generalizable to all patients with giant gastric GISTs. Further studies are needed to validate these results.

## Conclusions

This case demonstrates that giant posterior gastric GISTs can be successfully managed through organ-preserving wedge resection when careful patient selection and meticulous surgical technique are employed. Complete R0 resection without tumor rupture remains the cornerstone of treatment and may provide excellent oncologic and functional outcomes even in tumors exceeding 10 cm. In addition to achieving oncologic clearance, organ-preserving surgery may contribute to improved long-term quality of life and preservation of gastric function by maintaining normal gastric anatomy. Nevertheless, larger prospective studies are required to further validate the long-term oncologic safety and functional outcomes of wedge resection for giant gastric GISTs, particularly those exceeding 10 cm.
